# Factors associated with pathological complete remission after neoadjuvant chemoradiotherapy in locally advanced rectal cancer: a real-world clinical setting

**DOI:** 10.3389/fonc.2024.1421620

**Published:** 2024-08-07

**Authors:** Minglin Lin, Junsheng Liu, Chongyuan Lan, Ming Qiu, Wei Huang, Cun Liao, Sen Zhang

**Affiliations:** ^1^ Department of Colorectal and Anal Surgery, The First Affiliated Hospital of Guangxi Medical University, Nanning, China; ^2^ Guangxi Key Laboratory of Enhanced Recovery After Surgery for Gastrointestinal Cancer, Nanning, China

**Keywords:** rectal cancer, neoadjuvant chemoradiotherapy, pathological complete remission, tumor regression grade (TRG), clinical factors

## Abstract

**Objective:**

This study aims to identify factors associated with achieving a pathological complete remission (pCR) in patients with locally advanced rectal cancer (LARC) after neoadjuvant chemoradiotherapy (nCRT).

**Methods:**

We conducted a cohort analysis of 171 LARC patients who underwent curative resection post-nCRT at the First Affiliated Hospital of Guangxi Medical University between January 2015 and December 2021. The data encompassed clinical and pathological information. Univariate and binary logistic regression multivariate analyses were employed to examine the factors influencing pCR achievement after nCRT. Kappa value tests were utilized to compare clinical staging after nCRT with postoperative pathological staging.

**Results:**

Postoperative histopathology revealed that of the 171 patients, 40 (23.4%) achieved TRG 0 grade (pCR group), while 131 (76.6%) did not achieve pCR, comprising 36 TRG1, 42 TRG2, and 53 TRG3 cases. Univariate analysis indicated that younger age (*p*=0.008), reduced tumor occupation of intestinal circumference (*p* =0.008), specific pathological types (*p*=0.011), and lower pre-nCRT CEA levels (*p*=0.003) correlated with pCR attainment. Multivariate analysis identified these factors as independent predictors of pCR: younger age (OR=0.946, *p*=0.004), smaller tumor occupation of intestinal circumference (OR=2.809, *p*=0.046), non-mucinous adenocarcinoma pathological type (OR=10.405, *p*=0.029), and lower pre-nCRT serum CEA levels (OR=2.463, *p*=0.031). Clinical re-staging post-nCRT compared to postoperative pathological staging showed inconsistent MRI T staging (Kappa=0.012, *p*=0.718, consistency rate: 35.1%) and marginally consistent MRI N staging (Kappa=0.205, *p*=0.001, consistency rate: 59.6%).

**Conclusion:**

LARC patients with younger age, presenting with smaller tumor circumferences in the intestinal lumen, lower pre-nCRT serum CEA levels, and non-mucinous adenocarcinoma are more likely to achieve pCR after nCRT. The study highlights the need for improved accuracy in clinical re-staging assessments after nCRT in LARC.

## Introduction

Rectal cancer, a prevalent malignancy within the digestive tract, constitutes approximately 35% of colorectal cancer (CRC) (1–3). The International Agency for Research on Cancer (IARC) of the World Health Organization reports that CRC ranks third in global cancer incidence, following breast and lung cancers, and is second in mortality rates, trailing only lung cancer. In 2020, CRC accounted for roughly 1,931,590 new cases and about 935,173 deaths globally, representing 10.0% and 9.4% of all malignant tumor incidences and mortalities, respectively (4). The risk factors for CRC, once predominantly in high-income Western countries, such as poor diet, obesity, and sedentary lifestyles, are now also prevalent in some low- and middle-income regions (5). Lifestyle habits, including high consumption of animal-based foods and physical inactivity, significantly contribute to colorectal malignancy risks. Other factors include uncontrolled alcohol consumption, smoking, and high intake of red or processed meats. Conversely, moderate consumption of calcium, grains, fibers, and dairy products can mitigate the disease risk (6). The incidence and mortality of CRC vary globally, influenced by diverse exposure risks, demographic features, and genetics (7).

China has witnessed a surge in CRC incidence and mortality rates, attributed to socioeconomic advancements and shifts in lifestyle and dietary habits, leading to increased meat consumption and obesity (8, 9). CRC ranks third in incidence and fifth in mortality among all malignancies in China. Although rectal cancer forms part of CRC, its treatment modalities differ from those of colon cancer. Epidemiological studies indicate a higher postoperative recurrence rate for rectal cancer compared to colon cancer (10). Many patients are diagnosed with locally advanced rectal cancer (LARC) due to the subtlety of early symptoms. The definition of LARC varies: rectal cancer within 12 cm of the anus, extending beyond the muscular layer or involving mesorectal/true pelvis lymph nodes (c/pT3-4b, c/pN1-2), but without distant metastasis (11).

Surgery remains the cornerstone of advanced rectal cancer treatment, with total mesorectal excision (TME) established as the standard procedure since the 1980s (12). TME aims to remove the tumor completely while preserving the rectal sphincter and bowel function. However, due to the rectum’s complex anatomical location, surgery alone often falls short in efficacy. LARC poses surgical challenges, high complication rates, increased local recurrence rates, and lower rates of anal preservation, adversely affecting patients’ quality of life. The focus has shifted to strategies that reduce tumor size and staging, facilitate complete surgical resection and sphincter preservation, and minimize distant metastasis and local recurrence chances. Multi-disciplinary treatment (MDT) has emerged as a solution to these challenges. Neoadjuvant chemoradiotherapy (nCRT) precedes surgical resection and typically involves chemotherapy, targeted therapy, and local radiotherapy.

Following the National Cancer Institute’s consensus on colorectal cancer treatment in the 1990s, the standard approach for LARC has been radical resection followed by adjuvant radiochemotherapy (13). German studies confirmed the superiority of preoperative over postoperative radiochemotherapy in improving local control and sphincter preservation (14). Thus, since Rolf Sauer et al.’s 2004 publication, the consensus for LARC treatment in most countries has been nCRT combined with TME and postoperative adjuvant chemotherapy, often referred to as the “sandwich biscuit model” (15).

The LARC treatment model has evolved over the past decades. Neoadjuvant chemoradiotherapy, combined with TME, has proven effective in reducing local recurrence and is recommended by various cancer medical associations. Studies indicate nCRT’s efficacy in reducing tumor size and staging, enhancing sphincter preservation rates, and enabling some patients to achieve pathological complete remission (pCR) (16, 17). Patients achieving pCR reportedly have lower local recurrence rates and improved survival compared to non-pCR patients. However, not all patients benefit from nCRT, with some showing progression or insensitivity to treatment, leading to surgical delays and missed optimal treatment timings (18). Accurate assessment of cCR is critical to avoid overtreatment and unnecessary surgical trauma in patients achieving this response. However, the challenge lies in the accurate preoperative prediction of cCR, as cCR and pCR are not always congruent (19–21). Research into factors related to pCR is crucial for predicting prognosis, developing personalized treatment plans, and increasing pCR rates, thereby avoiding unnecessary surgery or missed surgical opportunities.

Given this context, our study undertakes a real-world clinical setting of patients undergoing TME after nCRT at the Colorectal and Anal Surgery Department of the First Affiliated Hospital of Guangxi Medical University. We explore various factors, including patients’ conditions, tumor characteristics, tumor markers, tumor immune-inflammatory microenvironment, and treatment methods, to predict prognosis and develop individualized treatment strategies for achieving pCR post-nCRT in LARC.

## Data and methods

### Study population and data collection

This study was approved by the institutional ethics review committee at the First Affiliated Hospital of Guangxi Medical University (Approved no.:2024-E164-01), and informed consents were obtained from each participant according to the committee’s regulations. This cohort study analyzed the clinical and pathological data of 210 patients diagnosed with locally advanced rectal cancer who underwent neoadjuvant chemoradiotherapy followed by surgical intervention at the Colorectal and Anal Surgery Department of the First Affiliated Hospital of Guangxi Medical University from January 2015 to December 2021. The treatment efficacy after neoadjuvant therapy was evaluated using the Response Evaluation Criteria in Solid Tumors. Exclusions were made for 8 cases classified as clinical complete response, 9 cases with distant metastasis, and 22 cases who were ineligible for surgery due to local tumor progression or other factors, resulting in 171 patients included in the final analysis, comprising 123 males and 48 females, with ages ranging from 20 to 77 years and a median age of 55 years.

### Inclusion and exclusion criteria

The inclusion criteria were: (1) Tumor lower edge within 12 cm from the anus as confirmed by rectal digital examination or colonoscopy; (2) Postoperative pathological confirmation of adenocarcinoma; (3) MRI evaluations pre- and post-neoadjuvant chemoradiotherapy (nCRT), with a clinical staging of LARC (T3-4b or N+); (4) No evidence of distant metastasis in pelvic-abdominal-thoracic CT scans and during surgery; (5) Completion of nCRT followed by total mesorectal excision (TME) surgery; (6) Comprehensive clinical and pathological data. The exclusion criteria were: (1) Presence of distant metastasis identified preoperatively or intraoperatively; (2) Incomplete nCRT; (3) Failure to undergo TME surgery; (4) Incomplete clinical and pathological data.

### Observational parameters

Parameters observed included gender, age, ethnicity, body mass index (BMI), smoking and alcohol consumption status, presence of diabetes, hypertension, anemia, serum albumin levels, pathological type, degree of differentiation, clinical T and N staging pre- and post-nCRT, circumferential resection margin (CRM), extramural venous invasion (EMVI), distance from the tumor lower edge to the anal margin, tumor circumference within the intestinal lumen, serum tumor markers (CEA, CA199) pre- and post-nCRT, interval between the end of radiotherapy and surgery, and pre-nCRT inflammatory factors (NLR, PLR, LMR), amounting to 30 potential indicators for predicting pCR in rectal cancer patients post-nCRT.

### Definitions of key indicators

The definitions for key indicators were as follows: Diabetes was defined based on either a prior diagnosis of diabetes or blood sugar levels meeting national diabetes diagnostic criteria during hospitalization; Hypertension was determined by a documented history of hypertension or blood pressure readings meeting the national hypertension diagnostic criteria during hospital stay; Smoking and drinking history referred to a past history of tobacco and alcohol use; Anemia was defined as hemoglobin in venous blood <110 g/L; The distance of the tumor lower edge from the anal margin was assessed using rectal digital examination, colonoscopy, and rectal MRI; TNM staging was based on clinical assessments using rectal MRI, endorectal ultrasound, and chest-abdominal-pelvic CT, reviewed by two qualified physicians; Pathological specimens were processed according to standard colorectal cancer specimen handling guidelines and analyzed by two qualified pathologists. Staging adhered to the American Joint Committee on Cancer/International Union Against Cancer (UICC) CRC TNM staging system (8th edition, 2017). CRM positivity was diagnosed using high-resolution MRI when tumors and metastatic lymph nodes were less than 1 mm from the mesorectal fascia (MRF) or adjacent organ structures. EMVI was diagnosed when high-resolution MRI showed extramural blood vessels infiltrated by the tumor beyond the rectal wall’s muscular layer. Tumor circumference in the intestinal lumen was assessed using colonoscopy, rectal MRI, and endorectal ultrasound. Tumor marker levels pre- and post-nCRT were measured within two weeks before starting nCRT and two weeks before surgery. Inflammatory indicators included NLR (neutrophil-to-lymphocyte ratio), PLR (platelet-to-lymphocyte ratio), and LMR (lymphocyte-to-monocyte ratio), with all biochemical and blood cell assessments conducted at the institutional laboratory according to standardized procedures.

### Treatment protocol

The standard long-course radiotherapy regimen was administered to all patients, entailing a total radiation dose of 45-50.4 Gy, administered at 1.8-2.0 Gy per fraction, five sessions per week, one session per day, with two rest days, culminating in 25-28 sessions over 5-6 weeks. Concurrently with radiotherapy, oral capecitabine tablets (825 mg/m², twice daily, 5 days per week) were prescribed. Following radiotherapy completion, the patients received two cycles consolidation chemotherapy. Then the patients underwent response evaluation by digital rectal examination, transrectal ultrasound, CT, and MRI. If it was able to achieve R0 resection, then patients underwent total mesorectal excision; If not, another two cycles chemotherapy were recommended. For those to pursuit pCR, six cycles chemotherapy were administered, each cycle lasting 21 days, consisting of oxaliplatin (130 mg/m², intravenous infusion, every 21 days) and capecitabine (825 mg/m², twice daily, days 1-14). Post-nCRT, patients underwent total mesorectal excision (TME) surgery, with the interval from the end of radiotherapy to surgery ranging from 6 to 37 weeks, and a median of 9 weeks.

### Neoadjuvant chemoradiotherapy efficacy assessment

Pathological complete remission (pCR) was defined as the absence of residual tumor cells under the microscope in post-TME surgery specimens, including lymph nodes, in locally advanced rectal cancer patients after nCRT (postoperative pathological staging pT0N0). The Ryan/American Joint Committee on Cancer (AJCC) 7th edition tumor regression grading (TRG) system was employed for assessment, categorizing responses based on the quantity of viable tumor cells and the degree of tissue fibrosis in pathological specimens; Grade 0 (complete regression): absence of residual tumor cells; Grade 1 (moderate regression): presence of single or small clusters of residual tumor cells; Grade 2 (minor regression): residual tumor with substantial fibrotic stroma; Grade 3 (no regression): extensive tumor residue, with no or minimal tumor cell apoptosis. Grade 0 in TRG grading was classified as pCR. The post-neoadjuvant treatment efficacy was evaluated using RECIST 1.1 criteria: (1) CR: total disappearance of all target lesions, including pathological lymph nodes, which must reduce to less than 10 mm on the short axis; (2) PR: a minimum 30% reduction in the sum of diameters of all target lesions, relative to baseline sum diameters; (3) PD: a minimum 20% increase in the sum of diameters of all target lesions, with an absolute increase of at least 5 mm, or the emergence of one or more new lesions; (4) SD: changes insufficient to meet PR or PD criteria.

### Statistical analysis

Data analysis was performed using SPSS 24.0 software. Quantitative data fitting normal or near-normal distribution were compared between groups using the t-test. For skewed data, median (interquartile range) [M(P25-P75)] representation was used, with the Mann-Whitney U test applied. Count data were analyzed using Pearson’s chi-square test, continuity-corrected chi-square test, or Fisher’s exact test. Binary logistic regression was utilized to assess multiple predictive factors. The concordance between MRI T and N staging post-nCRT and postoperative pathological staging was evaluated using the kappa value test, with a *p*-value <0.05 considered statistically significant.

## Results

### Treatment outcomes

All 171 participants successfully completed neoadjuvant chemoradiotherapy and total mesorectal excision as per the study protocol. Histopathological examinations post-surgery revealed that 40 cases (23.4%) achieved TRG0 level (pathological complete remission, pCR group), while 131 cases (76.6%) were categorized in the non-pCR group, including 36 in TRG1, 42 in TRG2, and 53 in TRG3. Notably, 4 patients in the non-pCR group demonstrated complete eradication of the primary intestinal tumor, yet residual tumor cells were identified in dissected lymph nodes.

### Surgical details

In the pCR group (40 patients), all underwent the Dixon procedure. Among the 131 patients in the non-pCR group, 101 underwent the Dixon procedure, 28 the Miles procedure, and 2 the Hartmann procedure. The overall sphincter preservation rate was 83.6% (143/171), with 100% in the pCR group. The R0 resection rate was uniformly 100% across both groups. Postoperative complications were observed in 28 cases (16%), with wound healing issues predominating (12 cases, 32% in the Miles group).

### Before neoadjuvant chemoradiotherapy T & N staging vs. postoperative pathological T & N staging

A statistically significant difference (P < 0.05) was observed when comparing pre-nCRT T staging with postoperative pathological T staging, suggesting nCRT’s effectiveness in reducing T stage (**Table 1**). Similarly, a statistically significant correlation (P < 0.05) was noted between pre-nCRT N staging and postoperative pathological N staging, affirming the efficacy of nCRT in reducing N staging (**Table 2**).

**Table 1:** Primary tumor (T) stage before nCRT and after surgery.

T stage (n)						χ^2^	P
	T0	T1	T2	T3	T4		
Before nCRT	0	0	2	121	48	152.276	0.001
After surgery	44	16	38	73	0	

**Table 2:** Regional lymph nodes (N) stage before nCRT and after surgery.

	N stage (n)			χ^2^	P
	N0	N1	N2		
Before nCRT	4	53	114	211.465	0.001
After surgery	130	31	10	

### EMVI and CRM before and after neoadjuvant chemoradiotherapy

A significant reduction (P < 0.05) in patients with positive extramural venous invasion (EMVI) and circumferential resection margin (CRM) was noted following nCRT (**Table 3**).

**Table 3:** Comparison of EMVI and CRM before and after nCRT.

	EMVI		CRM	
	Negative	Positive	Negative	Positive
Before nCRT	93	78	58	113
After nCRT	123	48	94	77
*χ^2^*	11.31		15.347	
*P*	0.01		0.001	

### CEA levels before and after neoadjuvant chemoradiotherapy

A statistically significant decrease (P < 0.05) in carcinoembryonic antigen (CEA) levels was observed post-nCRT, indicating nCRT’s capacity to lower CEA levels (**Table 4**).

**Table 4:** Comparison of CEA level before and after nCRT.

	CEA (ng/ml)	*z*	*P*
Before nCRT	5.21(2.41-13.59)	7.650	0.001
After nCRT	2.32(1.62-3.60)		

### MRI T & N staging consistency after neoadjuvant chemoradiotherapy and postoperative pathological T & N staging

An inconsistency between MRI T staging post-nCRT and postoperative pathological T staging was found (Kappa = 0.012, *p*= 0.718), with a concordance rate of 35.1% (60/171) (**Table 5**). Meanwhile, MRI N staging post-nCRT showed consistency with postoperative pathological N staging, albeit with poor congruence (Kappa = 0.205, *p*= 0.001), and a concordance rate of 59.6% (102/171) (**Table 6**).

**Table 5:** Consistency of primary tumor (T) stage between MRI staging and pathological staging after nCRT.

Pathological staging	MRI T stage (n)					Total
	T0	T1	T2	T3	T4	
T0	2	2	4	33	3	44
T1	0	0	3	12	1	16
T2	0	0	5	30	3	38
T3	0	0	3	53	17	73
T4	0	0	0	0	0	0
Total	2	2	15	128	24	171

**Table 6:** Consistency of regional lymph nodes (N) stage between MRI staging and pathological staging after nCRT.

Pathological staging	MRI N stage (n)		Total
	N0	N1-2	
N0	72	58	130
N1-2	11	30	41
Total	83	88	171

### Pathological complete remission and clinical pathology

Univariate analysis revealed no significant association between pCR post-nCRT and factors such as gender, ethnicity, BMI, smoking status, alcohol consumption, hypertension, diabetes, pre-nCRT anemia, albumin level, inflammatory markers (NLR, LMR, PLR), CA19-9 levels, and T/N staging pre- and post-nCRT. However, younger age, smaller tumor circumference in the intestinal lumen, non-mucinous adenocarcinoma pathological type, and lower pre-nCRT CEA levels were significantly related to achieving pCR (**Table 7**). Multivariate analysis identified younger age (OR=0.946, *p*=0.004), smaller tumor circumference in the intestinal lumen (OR=2.809, *p*=0.046), non-mucinous adenocarcinoma pathological type (OR=10.405, *p*=0.029), and lower pre-nCRT serum CEA levels (OR=2.463, *p*=0.031) as independent predictors of achieving pCR following nCRT in locally advanced rectal cancer (**Table 8**).

**Table 7:** Univariate analysis of pCR and clinicopathological factors.

Variable	pCR group (n=40)	Not pCR group (n=131)	*χ^2^/z*	*P*
Age/y	51(42-58)	56(48-62)	-2.662	0.008
Sex			0.802	0.37
Male	31	92		
Female	9	39		
Nationality			0.423	0.515
The Han	27	81		
other	13	50		
BMI			0.541	0.462
≤24 kg/m^2^	27	80		
>24kg/m^2^	13	51		
Smoking			1.061	0.303
No	23	87		
Yes	17	44		
Drink alcohol			2.252	0.133
No	22	89		
Yes	18	42		
Hypertension.			0.572	0.449
No	35	108		
Yes	5	23		
Diabetes.			0.932	0.334
No	37	126		
Yes	3	5		
Hemoglobin before nCRT			0.737	0.391
≥110g/L	35	107		
<110g/L	5	24		
Albumin before nCRT			0.534	0.465
≥40 g/L	24	70		
<40 g/L	16	64		
PLR before nCRT	130(106-184)	146(108-187)	0.405	0.685
NLR before nCRT	1.9(1.6-2.8)	2.1(1.5-2.7)	0.117	0.907
LMR before nCRT	3.5(2.8-5.0)	3.4(2.6-4.3)	1.335	0.182
CEA before nCRT			8.601	0.003
≤5ng/ml	28	57		
>5ng/ml	12	74		
CA199 before nCRT			0.659	0.417
≤37U/ml	30	106		
>37U/ml	10	25		
CEA after nCRT			1.108	0.293
≤5ng/ml	37	113		
>5ng/ml	3	18		
CA199 after nCRT			0.068	0.794
≤37 U/ml	38	123		
>37 U/ml	2	8		
T category before nCRT			0.802	0.370
T3	31	92		
T4	9	39		
N category before nCRT			1.044	0.307
N0-1	16	41		
N2	24	90		
CRM before nCRT			1.716	0.190
Negative	17	41		
Positive	23	90		
nCRT before EMVI			3.620	0.057
Negative	27	66		
Positive	13	65	0.621	
Distance of tumor from anal margin				0.431
≤5cm	14	55		
>5cm	26	76		
Tumor circumluminal diameter			7.050	0.008
<1	34	82		
=1	6	49		
Pathological type			6.537	0.011
Non-mucinous adenocarcinoma	39	106		
Mucinous adenocarcinoma	1	25		
Degree of differentiation			0.054	0.974
Low	4	14		
Middle	33	106		
High	3	11		
T category after nCRT			5.015	0.069
T0-2	8	11		
T3	29	99		
T4	3	21		
N category after nCRT			3.649	0.161
0	22	61		
1	15	43		
2	3	27		
CRM after nCRT			3.311	0.069
Negative	27	67		
Positive	13	64		
EMVI after nCRT			4.418	0.036
Negative	34	89		
Positive	6	42		
Interval time from the end of radiotherapy to operation			0.541	0.462
>8 weeks	27	80		
≤8 weeks	13	51		

**Table 8:** Multivariate analysis of pCR after nCRT for rectal cancer.

Variable	*b* value	SE value	Wald value	*OR*	95%CI	*P* value
Age	0.056	0.019	8.311	0.946	0.911-0.982	0.004
CEA before nCRT	0.901	0.419	4.638	2.463	1.084-5.594	0.031
Tumor occupies the proportion of intestinal cavity	1.033	0.518	3.975	2.809	1.018-7.756	0.046
EMVI after nCRT	0.964	0.509	3.584	2.622	0.967-7.110	0.058
Pathological type	2.342	1.073	4.766	10.405	1.270-85.208	0.029

## Discussion

The prevailing standard treatment for locally advanced rectal cancer (LARC) encompasses a comprehensive approach that integrates neoadjuvant chemoradiotherapy (nCRT), total mesorectal excision (TME) surgery, and postoperative adjuvant chemotherapy. Pioneering research by Rolf Sauer et al. from the German Rectal Cancer Study Group established that preoperative chemoradiotherapy surpasses postoperative chemoradiotherapy in enhancing local control rates and improving sphincter preservation opportunities (14, 15). Further literature supports nCRT’s role in shrinking tumor size and stage, sometimes rendering originally inoperable tumors resectable, along with increasing rates of sphincter preservation (22). Notably, a proportion of patients attain a pCR post-nCRT (16, 17). Our study reinforces these findings, evidencing the efficacy of nCRT in downstaging LARC (*p*<0.05) and highlighting its pivotal role in improving local tumor staging.

The study also notes that LARC patients who achieve pCR post-nCRT tend to have a more favorable long-term prognosis compared to those with residual tumor pathology in postoperative specimens. The “Watch and Wait” (W&W) strategy, an alternative to radical resection, has gained increasing attention over the past decade. This approach involves close monitoring of patients who attain clinical complete response (cCR) post-nCRT without immediate surgical intervention, providing salvage surgery upon recurrence. Standard clinical reassessment post-nCRT includes rectal digital examination, endoscopy, and MRI. However, due to current assessment limitations, cCR does not invariably equate to pCR, with some patients clinically classified as cCR demonstrating up to 50% tumor residue postoperatively (20, 21). Furthermore, a subset of patients may experience complete primary tumor disappearance but still harbor residual lymph node metastases (23). In our study, 9% (4/44) of patients exhibited complete eradication of the primary intestinal tumor, yet tumor cells persisted in dissected lymph nodes. This underscores the need for accurate preoperative pCR predictive markers to safely identify potential W&W candidates. Prior research has highlighted several factors correlated with pCR, including lower tumor grading, lower clinical T and N staging, higher radiation doses, and delayed surgery post-radiotherapy (24–26). Identifying these predictive factors preoperatively holds clinical significance for optimizing pCR achievement and selecting patients who might forego surgery post-nCRT.

The reported pCR rates post-neoadjuvant treatment for rectal cancer showed considerable variation in the literature, both nationally and globally. Studies from Denmark, South Korea, Switzerland, and the USA report pCR rates ranging from 13.8% to 60% (24, 27). In China, Liu Qizhi et al. reported a 29% pCR rate, while Sha Yingjiao et al. noted a 17.3% rate (28, 29). This variability likely relates to differences in population demographics, regional factors, and lifestyle habits. In our study, 23.4% of patients undergoing nCRT followed by TME achieved pCR, aligning with these global trends. Multivariate analysis identified age, pathological type, tumor circumference in the intestinal lumen, and pre-nCRT CEA levels as independent predictors for pCR in LARC post-nCRT.

Retrospective analyses have identified that mucinous tumors, poor differentiation, and the presence of extensive ulceration are associated with a diminished response to neoadjuvant chemoradiotherapy (nCRT) (30, 31). Colorectal cancer encompasses a diverse range of histological types. The WHO’s classification of digestive system tumors (4th edition) categorizes adenocarcinomas into subtypes including tubular, mucinous, signet ring cell, serrated, micropapillary, medullary, and cribriform comedo-type adenocarcinomas. Mucinous adenocarcinoma, as defined by WHO, is characterized by tumors in which over 50% of the glandular cells produce mucus, constituting approximately 2.6%-24.5% of colorectal malignancies (32). Signet ring cell carcinoma, marked by copious mucus secretion and predominantly composed of signet ring cells, is comparatively rare in colorectal cancer. Studies have demonstrated that mucinous and signet ring cell carcinomas exhibit a poorer prognosis relative to papillary and tubular adenocarcinomas (33). A retrospective analysis by researchers at the Digestive Disease Research Institute of Cleveland Clinic, involving 306 stage II or III rectal cancer patients who underwent nCRT followed by surgery, suggested a correlation between tumor type and differentiation degree with the incidence of pCR (24). Molavi et al.’s research posits that the highly invasive nature of mucinous tumor cells may contribute to their lower responsiveness to nCRT (34). Our study concurs with these findings, revealing a significantly lower pCR rate in patients with mucinous adenocarcinoma post-nCRT.

CEA, a high-molecular-weight glycoprotein belonging to the immunoglobulin family, is detectable in biopsy samples and typically identified in serum, where it is normally below 5 ng/ml in adults. Environmental factors like smoking, viral infections, colitis, pancreatitis, and cirrhosis can influence CEA expression. Over the years, CEA has served as a biomarker for colon cancer and other tissue cancers (35). It is effective in diagnosing cancers of the intestine, pancreas, stomach, breast, thyroid, and ovaries, with its highest expression usually not exceeding 20 ng/ml (36). Only about 30-50% of colorectal cancer patients exhibit elevated CEA levels at initial diagnosis, limiting its sensitivity and reliability for early CRC detection (37). The primary utility of CEA testing lies in cancer progression monitoring; continuous preoperative and postoperative serum CEA measurements assist in predicting cancer staging, progression, and recurrence post-treatment. Like most tumor markers, serum CEA levels increase with disease progression. Serum CEA is also pivotal in monitoring colorectal cancer treatment effectiveness; successful therapy should normalize serum CEA levels, and a post-treatment increase often indicates tumor recurrence (38). Earlier studies comparing tumor marker levels pre- and post-nCRT identified a reduction in CEA levels post-nCRT, underscoring its significance in reflecting nCRT efficacy for rectal cancer (39). This study also reports a significant decrease in serum CEA levels following nCRT (P<0.05), aligning with prior findings and indicating that nCRT effectively lowers CEA levels.

Compared with CEA, circulating tumor DNA (ctDNA), a small DNA fragment derived from tumor cells, presents an emerging molecular marker in cancer treatment and monitoring. Research indicates that ctDNA can reflect tumor genomic changes and has significant advantages in early evaluation of treatment response and prediction of recurrence (40). The ability of ctDNA to detect microscopic residual disease following curative-intent therapy for patients with early-stage colorectal cancer could enhance individualized adjuvant therapy and improve prognosis (41). Particularly after nCRT, a decrease in ctDNA levels is associated with pathological complete remission (pCR), suggesting that ctDNA may be a valuable predictor and monitoring tool (42, 43). ctDNA can detect disease recurrence several months prior to imaging, providing an opportunity for early intervention with a potential impact on survival (44). Regular postoperative ctDNA level testing can detect residual disease or recurrence early, providing opportunities for timely intervention. Integration of ctDNA analysis into clinical practice could revolutionize colorectal cancer management by enabling earlier interventions, tailoring therapies to individual genetic profiles, and improving overall survival outcomes.

The optimal timing of surgery for LARC following nCRT requires balancing maximal post-radiotherapy effects against the risk of acute tissue adverse reactions or tumor recurrence, ensuring surgical safety. Tumor response to nCRT is time-dependent, occasionally requiring months to achieve maximal tumor regression (45). Conventionally, the recommended interval from nCRT completion to surgery is 6-8 weeks, maximizing tissue response and mitigating radiation-related adverse effects. Under the “Watch and Wait” strategy, the ideal interval between treatment completion and surgery is designed to achieve the best balance between maximal tumor regression and treatment-induced complications or tumor recurrence, thus enhancing pCR chances and ensuring safety for necessary surgeries. Internationally, the suggested interval for long-course nCRT patients is 6-8 weeks (ESMO guidelines) or 5-12 weeks (NCCN guidelines). The current interval in China is 5-12 weeks, yet there is no consensus on the ideal interval post-nCRT. Radiation-induced necrosis requires time to develop, and an extended interval between radiation and surgery could increase the risk of organ function damage and complications. Studies have shown that delaying surgery beyond the typical 6-8 weeks results in a higher pCR rate without increasing surgical complications (46–48). Donlin et al.’s meta-analysis of 19,652 patients found significantly increased pCR values in LARC patients with an interval over 8 weeks post-radiotherapy compared to those with shorter intervals, with no notable differences in surgery duration, postoperative complications, or sphincter-preserving surgeries (26). A meta-analysis indicated that preoperative long-course chemoradiotherapy with delayed surgery significantly improved tumor downstaging, R0 resection, and pCR rates compared to preoperative short-course radiotherapy, without significantly impacting overall postoperative complications, despite increased acute side effects. These findings suggest that extending the waiting time beyond 8 weeks increases pCR probability in LARC patients, without significant differences in postoperative complications and sphincter-preserving surgeries. However, this study found no statistical difference in the interval between radiotherapy and surgery in terms of pCR (*p*>0.05), possibly due to its retrospective nature, small sample size, multiple confounding factors, and partial data loss, highlighting the need for further prospective research.

The RAPIDO and PRODIGE 23 trials have significantly advanced the field of rectal cancer treatment by demonstrating the efficacy of total neoadjuvant treatment (TNT) (49, 50). The RAPIDO trial showed that integrating short-course radiotherapy followed by chemotherapy before surgery markedly improved disease-related treatment failure rates and reduced the incidence of distant metastases. Similarly, the PRODIGE 23 trial highlighted the benefits of incorporating FOLFIRINOX into the neoadjuvant regimen, resulting in higher pathological complete response rates and enhanced overall survival. These landmark studies highlight the potential of TNT to transform rectal cancer management by optimizing preoperative therapy to target systemic disease more effectively and improve long-term outcomes. The incorporation of these findings offers a robust framework for refining treatment strategies and personalizing patient care in rectal cancer.

This study is subject to certain limitations: firstly, it is a single-center study, prone to a significant rate of clinical data loss, which weakens the evidence level; secondly, the patient cohort is relatively small; thirdly, the assessment indicators are not exhaustive, and there is a lack of comparative analysis with other radiotherapy and chemotherapy regimens.

In conclusion, neoadjuvant chemoradiotherapy demonstrates efficacy in reducing tumor staging and levels of the tumor marker CEA, with some patients achieving pCR post-nCRT. This study successfully identifies younger age, smaller tumor circumference, non-mucinous adenocarcinoma pathological type, and lower pre-nCRT CEA levels as independent predictors of pCR. However, the precision of clinical staging assessment following nCRT is currently hindered by limitations in existing technologies, resulting in discrepancies between cCR and pCR. Consequently, there is a need for further comprehensive research with more varied samples, extensive data, innovative assessment indicators, and multi-center prospective studies.

## Data availability statement

The original contributions presented in the study are included in the article/supplementary material. Further inquiries can be directed to the corresponding author.

## Ethics statement

The studies involving humans were approved by The Medical Ethics Committee of First Affiliated Hospital of Guangxi Medical University. The studies were conducted in accordance with the local legislation and institutional requirements. Written informed consent for participation in this study was provided by the participants’ legal guardians/next of kin.

## Author contributions

ML: Validation, Funding acquisition, Writing – review & editing, Writing – original draft, Methodology, Investigation. JL: Writing – original draft, Software, Methodology, Investigation, Data curation. CL: Writing – original draft, Formal analysis, Data curation. MQ: Writing – review & editing, Software, Data curation. WH: Writing – review & editing, Formal analysis. CL: Writing – review & editing, Visualization, Resources, Data curation. SZ: Writing – review & editing, Validation, Supervision, Project administration.
